# p*K*_a_ of the ligand water molecules in the oxygen-evolving Mn_4_CaO_5_ cluster in photosystem II

**DOI:** 10.1038/s42004-020-00336-7

**Published:** 2020-07-16

**Authors:** Keisuke Saito, Minesato Nakagawa, Hiroshi Ishikita

**Affiliations:** 1 Department of Applied Chemistry, The University of Tokyo, 7-3-1 Hongo, Bunkyo-ku, Tokyo 113-8654 Japan; 2 Research Center for Advanced Science and Technology, The University of Tokyo, 4-6-1 Komaba, Meguro-ku, Tokyo 153-8904 Japan

**Keywords:** Metalloproteins, Enzyme mechanisms, X-ray crystallography

## Abstract

Release of the protons from the substrate water molecules is prerequisite for O_2_ evolution in photosystem II (PSII). Proton-releasing water molecules with low p*K*_a_ values at the catalytic moiety can be the substrate water molecules. In some studies, one of the ligand water molecules, W2, is regarded as OH^−^. However, the PSII crystal structure shows neither proton acceptor nor proton-transfer pathway for W2, which is not consistent with the assumption of W2 = OH^−^. Here we report the p*K*_a_ values of the four ligand water molecules, W1 and W2 at Mn4 and W3 and W4 at Ca^2+^, of the Mn_4_CaO_5_ cluster. p*K*_a_(W1) ≈ p*K*_a_(W2) << p*K*_a_(W3) ≈ p*K*_a_(W4) in the Mn_4_CaO_5_ cluster in water. However, p*K*_a_(W1) ≈ p*K*_a_(D1-Asp61) << p*K*_a_(W2) in the PSII protein environment. These results suggest that in PSII, deprotonation of W2 is energetically disfavored as far as W1 exists.

## Abstract (web summary)

Water ligands found in the oxygen-evolving Mn_4_CaO_5_ cluster in photosystem II are thought to be important during photosynthesis, but the nature of the proton-releasing substrate water molecules is disputed. Here the pKa values of four key water molecules are estimated, with implications for the mechanism of water oxidation.

## Introduction

**Fig. 1:**
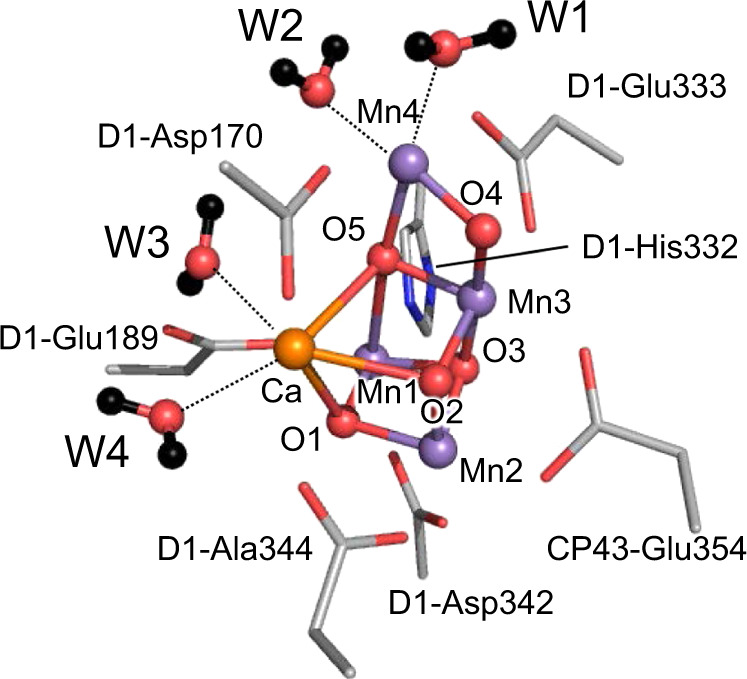
Structure of the Mn_4_CaO_5_ cluster. The second sphere ligand residues (D1-Asp61 and CP43-Arg357) are not shown. Dotted lines indicate ligations of the ligand water molecules to the Mn4 and Ca^2+^ sites.

In the water-splitting enzyme, photosystem II (PSII), oxygen evolution proceeds, removing four protons (H^+^) and four electrons from two substrate water molecules at the oxygen-evolving complex, Mn_4_CaO_5_ (Fig. 1)^1,2^. The Mn_4_CaO_5_ cluster has two ligand water molecules, W1 and W2, at the dangling Mn4 site and another two ligand water molecules, W3 and W4, at the Ca^2+^ site. These bound water molecules are candidates as potential substrates for water oxidation. As electron transfer occurs, the oxidation state of the oxygen-evolving complex, S_*n*_, increases. Release of protons is observed with the typical stoichiometry of 1:0:1:2 for S_0_ → S_1_ → S_2_ → S_3_ → S_0_, and O_2_ is evolved in the S_3_ to S_0_ transition. After O_2_ evolution, the first proton-releasing step is the S_0_ to S_1_ transition. The Mn_4_CaO_5_ cluster has a chain of strongly H-bonded 8 water molecules (O4-water chain) directly linked to O4 (linking Mn4 and Mn3 in the Mn_3_CaO_4_-cubane) and the release of the proton occurs along the O4-water chain in the S_0_ to S_1_ transition^3–5^. In S_0_ and S_1_^6^, the ligand water molecules W1–W4 are H_2_O in quantum mechanical/molecular mechanical (QM/MM) models (i.e., in the presence of the PSII protein environment)^3,7,8^, whereas W2 is assumed to be OH^−^ in simplified QM models (i.e., in the absence of the PSII protein environment)^9–11^. The release of the proton is also observed in the S_2_ to S_3_ transition. Based on the observations of the recent radiation-damage-free structures obtained using the X-ray free electron laser (XFEL), the sixth O site, O6, may be incorporated into the Mn1 and O5 moieties of the Mn_4_CaO_5_cluster in the S_2_ to S_3_ transition^12–14^.

During the water incorporation process, deprotonation of the water molecule may occur as proposed (e.g., water incorporation into the Mn1 moiety^15,16^, water incorporation from the Ca^2+^ moiety^17–19^). In theoretical models by Shoji et al.^17^ and Isobe et al.^20^, it was assumed the presence of OH^−^ at W2 in S_2_ to facilitate the release of the proton from H_2_O at W3 to OH^−^ at W2, and proposed that deprotonation of W3 at the Ca^2+^ moiety occurs in the S_2_ to S_3_ transition. If OH^−^ needed to be located at either W2 or W3, placing OH^−^ at W2 and H_2_O at W3 would be consistent with the experimentally measured values, p*K*_a_(Ca^2+^) = 12.8 >> p*K*_a_(Mn^3+^) = 0.7^21^ in water. In theoretical models by Ames et al.^22^, Rapatskiy et al.^23^, Pérez-Navarro et al.^24^, and Capone et al.^25^, W2 was also assumed to be OH^–^.

If the Mn_4_CaO_5_ cluster were isolated from the protein environment, placing OH^−^ at W2, not at W3, might be explained by p*K*_a_(Ca^2+^) >> p*K*_a_(Mn^3+^). However, placing OH^−^ at W2 is not consistent with the PSII crystal structures. The PSII crystal structures show that W2 has no strong H-bond acceptor, whereas W1 has a strong H-bond acceptor, D1-Asp61. Quantum mechanical/ molecular mechanical calculations show that H_2_O at W1 forms a low-barrier H-bond with D1-Asp61 and is ready for proton transfer in S_2_^26^. This may correspond to the significant changes in the H-bond properties between D1-Asp61 and a water molecule in the S_1_ to S_2_ transition observed in Fourier transform infrared (FTIR) spectroscopy^27^. Consistently, FTIR spectra suggested that W2 is H_2_O in S_1_ and S_2_^8^.

As far as we are aware, the p*K*_a_ values of the four water molecules at the Mn_4_CaO_5_ moiety, even those for the ligand water molecules W1–W4 are not reported. Robertazzi et al. reported that in S_1_, p*K*_a_(W2) = 6.1 for the isolated Mn_4_CaO_5_ cluster with deprotonated D1-His337 and 7.8 for the isolated Mn_4_CaO_5_ cluster with protonated D1-His337 in water, based on quantum chemical calculations^28^. However, the p*K*_a_ values for W1, W3, and W4 are not reported, which prevent from identifying the deprotonation sites even in the isolated Mn_4_CaO_5_ cluster in water. It should also be noted that the definition of the Mn_4_CaO_5_ cluster is vague. It can be comprised of Mn_4_CaO_5_, four ligand water molecules (W1–W4), and seven ligand residues (D1-Asp170, D1-Glu189, D1-His332, D1-Glu333, D1-Asp342, D1-Ala344, and CP43-Glu354, Fig. 1). It can also include the second sphere ligand residues, D1-Asp61, and CP43-Arg357, or the O4-water chain that forms an H-bond with O4. D1-Asp61 serves as an H-bond acceptor for W1 and is likely to facilitate proton transfer in the S_2_ to S_3_ transition^26^. The O4-water chain forms a significantly short H-bond with O4 (O…O < 2.5 Å) in the crystal structures in S_1_ (or a slightly lower S-state)^29,30^ and facilitates the release of the proton from O4 in the S_0_ to S_1_ transition^3–5^. The involvement of these proton acceptor groups (i.e., proton transfer pathways) facilitates deprotonation of the H-bond donor sites of the Mn_4_CaO_5_ cluster and decreases the p*K*_a_ values. This fact already implies that the p*K*_a_ values of the isolated Mn_4_CaO_5_ cluster in water are far from the relevant p*K*_a_ values in the PSII protein environment.

Here we report the p*K*_a_ values of the W1–W4 sites in the isolated Mn_4_CaO_5_ cluster in water, using quantum chemical approaches. To investigate the energetics of release of the proton towards the proton-transfer pathways in PSII, we analyze the potential-energy profiles of the H-bonds between the ligand water molecules and the H-bond acceptor (i.e., the proton acceptor) groups in the PSII protein environment. The results show that p*K*_a_(W1) ≈ p*K*_a_(W2) << p*K*_a_(W3) ≈ p*K*_a_(W4) in the Mn_4_CaO_5_ cluster in water (i.e., in the absence of the PSII protein environment), whereas p*K*_a_(W1) ≈ p*K*_a_(D1-Asp61) << p*K*_a_(W2) in PSII.

## Results and discussion

**Fig. 2:**
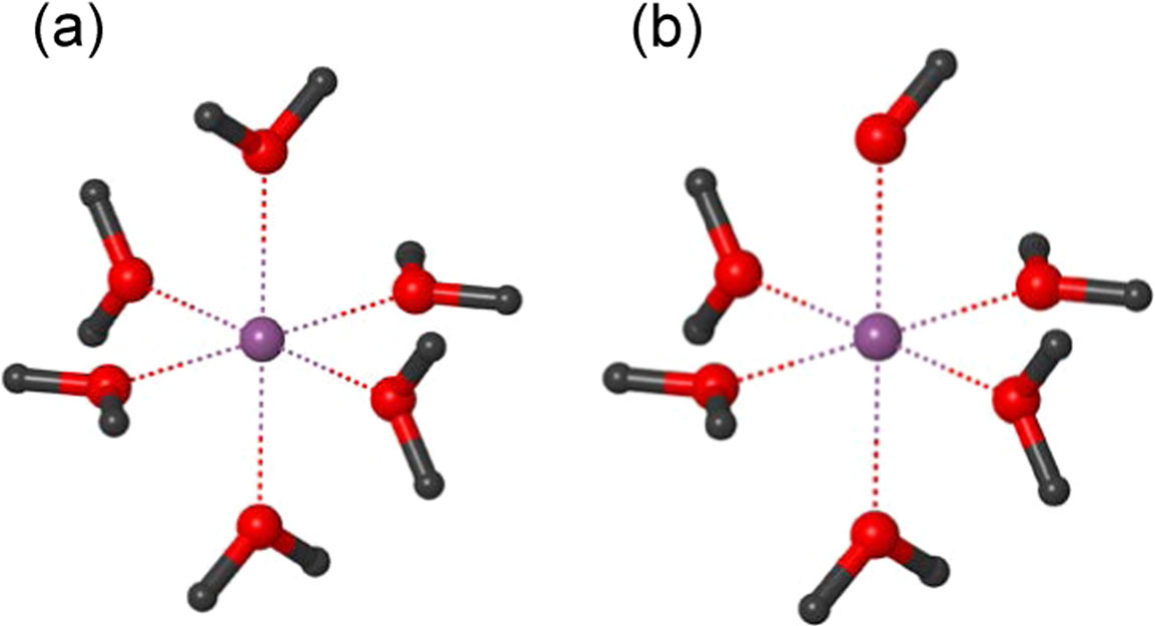
Structures of hexa-aqua metal complexes. **a** Protonated state. **b** Deprotonated state. Magenta balls indicate metal ions. Dotted lines indicate ligations of the ligand water molecules to metal ions.

**Fig. 3:**
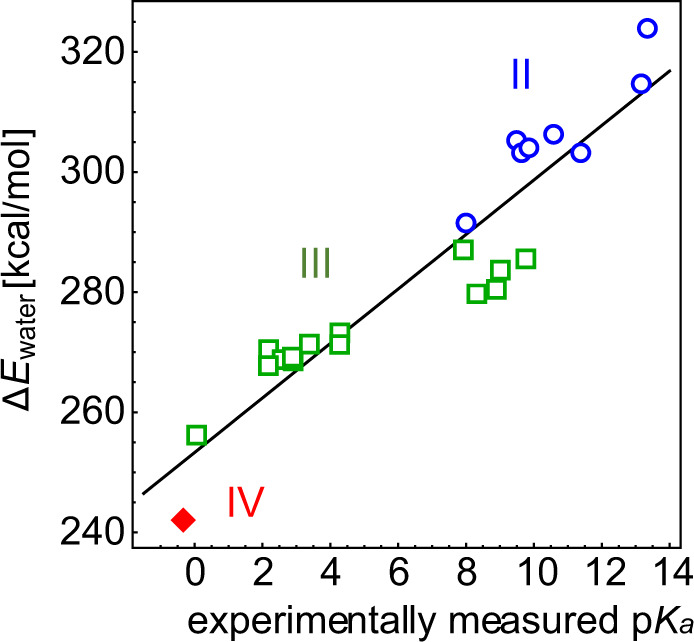
Experimentally measured p*K*_a_ values and calculated H_2_O/OH^−^ energy differences of hexa-aqua metal complexes. Blue open circles for divalent (II) metals, green open squares for trivalent (III) metals, and red closed diamond for the tetravalent (IV) metal.

We calculate the energy difference (Δ*E*_water_) between the protonated and deprotonated states of hexa-aqua metal complexes in water (Fig. 2). The calculated Δ*E*_water_ values of hexa-aqua metal complexes with the valences of II, III, and IV show a correlation with the experimentally measured p*K*_a_ values (Fig. 3) and are best fitted to the following equation:

1 $${\mathrm{p}}K_{\mathrm{a}} = 0.220\,\Delta E_{{\mathrm{water}}}\left[ {{\mathrm{kcal}}/{\mathrm{mol}}} \right]-55.8$$

The calculated p*K*_a_ values of hexa-aqua metal complexes obtained using Eq. 1 are listed in Table 1. Note that in vacuum, the experimentally measured p*K*_a_ values cannot be reproduced using a single equation (Supplementary Fig. 1, Supplementary Table 1), because the electrostatic influence between the cationic metal and anionic OH^−^ in the deprotonated state is overestimated in vacuum. Thus, p*K*_a_ predominantly depends on the metal valence in vacuum.

**Table 1:** Calculated (Calc.) and experimentally measured (Expl.) p*K*_a_ of hexa-aqua metal complexes.

Metal ion	Calc. p*K*_a_	Expl. p*K*_a_
Zr^4+^	−2.6	−0.32^a^
Mn^3+^ (high spin)	0.5	0.08^b^
Fe^3+^ (high spin)	3.1	2.19^b^
Ti^3+^	3.7	2.20^b^
V^3+^	3.3	2.60^b^
Ru^3+^ (low spin)	3.4	2.90^b^
Co^3+^ (low spin)	3.3	2.92^b^
Rh^3+^ (low spin)	3.9	3.40^b^
Cr^3+^	3.9	4.29^b^
Sc^3+^	4.3	4.30^b^
Lu^3+^	5.7	7.94^a^
Cu^2+^	10.9	8.0^b^
Y^3+^	7.0	8.34^a^
Pr^3+^	5.9	8.91^a^
La^3+^	7.3	9.03^a^
Fe^2+^ (high spin)	11.3	9.50^b^
Co^2+^ (high spin)	10.9	9.65^b^
Gd^3+^	6.6	9.78^a^
Ni^2+^	11.1	9.86^b^
Mn^2+^ (high spin)	11.6	10.59^b^
Mg^2+^	8.3	11.41^a^
Sr^2+^	13.4	13.18^a^
Ba^2+^	15.5	13.36^a^
RMSD^c^	1.5	

^a^Ref. ^21^.

^b^Ref. ^34^.

^c^Root mean square deviation (RMSD) of the calculated p*K*_a_ values from the experimentally measured p*K*_a_ values.

Calc. p*K*_a_ is obtained using Eq. (1).

The isolated Mn_4_CaO_5_ cluster is comprised of Mn_4_CaO_5_, four ligand water molecules (W1–W4), and seven ligand residues (D1-Asp170, D1-Glu189, D1-His332, D1-Glu333, D1-Asp342, D1-Ala344, and CP43-Glu354, Fig. 1). Using Eq. (1), the p*K*_a_ values for W1–W4 are calculated at the isolated Mn_4_CaO_5_ cluster in water (in the absence of the PSII protein environment, Fig. 1). p*K*_a_(W1) and p*K*_a_(W2) on the dangling Mn4 site are 7–11, whereas p*K*_a_(W3) and p*K*_a_(W4) on Ca^2+^ site are 14–18 (Table 2). p*K*_a_(W1) and p*K*_a_(W2) are higher than p*K*_a_ of 0.5 for Mn^3+^ (Table 1), because Mn4 has two ligand acidic residues, D1-Asp170 and D1-Glu333, and two μ-oxo O atoms, O4 and O5 (Fig. 1). The difference in the p*K*_a_ of >5 between the Mn4 and Ca^2+^ sites (Table 2) indicate that the Ca^2+^ site is originally disadvantageous for H_2_O deprotonation with respect to the Mn4 site. In particular in the PSII protein environment, W1 at Mn4 has D1-Asp61 as an H-bond acceptor, whereas W3 and W4 at Ca^2+^ do not have the corresponding acidic residues (see below). Thus, deprotonation of H_2_O and incorporation of the generated OH^–^ into the Mn_4_CaO_5_ cluster occurring at the Ca^2+^ moiety in the S_2_ to S_3_ transition (e.g., refs. ^17–19^) needs to overcome the energetic disadvantage.

**Table 2:** p*K*_a_ of the Mn_4_CaO_5_ cluster in water. Supplementary Table 2 for anti-ferromagnetic spin configuration.

S state	Mn1, Mn2, Mn3, Mn4^a^	W1	W2	W3	W4
S_0_	III, IV, III, III	11.3	10.1	17.7	16.4
S_0_ [O4-H]^b^	III, IV, III, III	9.5	9.0	16.6	16.0
S_0_ [O5-H]^c^	III, IV, III, III	10.0	8.5	16.1	15.4
S_1_	III, IV, IV, III	10.2	9.0	15.6	15.1
S_2_ [open]^d^	III, IV, IV, IV	8.3	8.2	15.6	15.9
S_2_ [closed]^e^	IV, IV, IV, III	9.7	7.1	14.2	15.6

^a^Ferromagnetic spin configuration.

^b^O4 is protonated.

^c^O5 is protonated.

^d^Open-cubane structure.

^e^Closed-cubane structure.

As far as the ligand coordination in the PSII protein structure is maintained (i.e., the torsion angles are fixed), p*K*_a_(W2) is only marginally (~1 p*K*_a_ unit) lower than p*K*_a_(W1) in the absence of the PSII protein environment (Table 2). When the geometry is fully relaxed (i.e., the torsion angles are not fixed) and OH^−^ is initially placed at W4 [to calculate p*K*_a_(W4)], proton transfer occurs from W2 via W3 to W4 occurs and OH^−^ is finally stabilized at W2, not at W1 (Supplementary Fig. 2). Deprotonation of W2 instead of W1 is just an artifact as W2, W3, and W4 form the H-bond network, pushing W1 and W3 away from Mn4 and Ca^2+^, respectively (W1...Mn4 = 3.8 Å and W3...Ca^2+^ = 3.6 Å). These observations may be a basis of why electron paramagnetic resonance (EPR) signals were often interpreted based on theoretical models, in which W2 was assumed to be OH^−^ in QM-based models (i.e., in the absence of the PSII protein environment, e.g., refs. ^10,22,23^). Interestingly, W2 = OH^−^ are also assumed in other QM-based models (e.g., by Siegbahn^9^ and Retegan et al.^11^), without using QM/MM approaches. It should be noted that W2, W3, and W4 never form the H-bond network as far as the PSII protein environment exists.

**Fig. 4:**
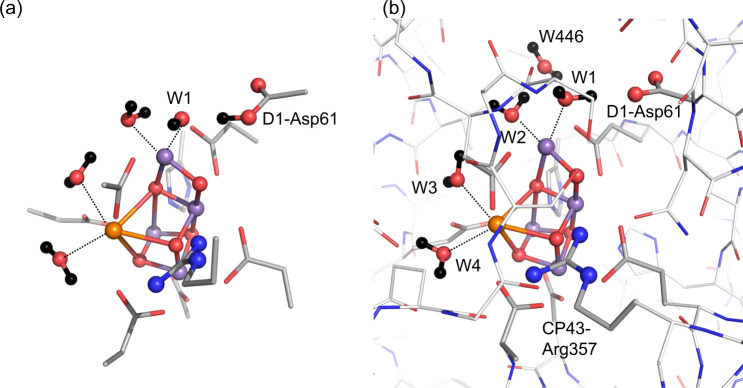
Mn_4_CaO_5_ structures including the second sphere ligand residues (D1-Asp61 and CP43-Arg357) in S_1_. Dotted lines indicate ligations of the ligand water molecules to metal ions. **a** Quantum-chemically optimized structure in the absence of the PSII protein environment. W1 is stabilized as OH^−^ in the presence of protonated D1-Asp61, as the release of the proton occurs from H_2_O at W1 to ionized D1-Asp61. **b** QM/MM-optimized Mn_4_CaO_5_ structure in the PSII protein environment.

The marginally low p*K*_a_(W2) with respect to p*K*_a_(W1) (Table 2) were the case only when the Mn_4_CaO_5_ cluster could be ideally isolated from the PSII protein environment. In such a model system, p*K*_a_(W1) and p*K*_a_(W2) can change easily, depending on even the definition of the Mn_4_CaO_5_ cluster. When the second sphere ligand residues (D1-Asp61 and CP43-Arg357) are included in the model system of the Mn_4_CaO_5_ cluster in water, H_2_O at W1 is not stable, releasing the proton, and is stabilized as OH^–^ at W1 in the presence of protonated D1-Asp61 (Fig. 4a), i.e., p*K*_a_(W1) << p*K*_a_(W2) (Table 3). The absence of the corresponding acidic residue as an H-bond acceptor for W2 and the proceeding proton transfer pathway (e.g., the D1-Asp61 pathway for W1^26^) contribute to an increase in p*K*_a_(W2) with respect to p*K*_a_(W1).

**Table 3:** p*K*_a_ of the Mn_4_CaO_5_ cluster with D1-Asp61 and CP43-Arg357 in water.

S state	Mn1,Mn2,Mn3,Mn4^a^	W1	W2	W3	W4
S_0_	III, IV, III, III	<<p*K*_a_(W2)^f^	10.6	17.4	15.7
S_0_ [O4H]^b^	III, IV, III, III	<<p*K*_a_(W2)^f^	9.6	15.5	14.8
S_0_ [O5H]^c^	III, IV, III, III	<<p*K*_a_(W2)^f^	8.8	14.9	14.7
S_1_	III, IV, IV, III	<<p*K*_a_(W2)^f^	10.2	16.6	13.6
S_2_ [open]^d^	III, IV, IV, IV	<<p*K*_a_(W2)^f^	9.1	13.8	13.8
S_2_ [closed]^e^	IV, IV, IV, III	<<p*K*_a_(W2)^f^	8.8	14.1	14.6

^a^Ferromagnetic spin configuration.

^b^O4 is protonated.

^c^O5 is protonated.

^d^Open-cubane structure.

^e^Closed-cubane structure.

^f^not determined because of deprotonation of W1 to D1-Asp61.

In the isolated Mn_4_CaO_5_ cluster in water (in the absence of the PSII protein environment), proton release occurs along the transiently formed H-bond between the ligand water molecule and a bulk water molecule (i.e., mobile water molecule with a high dielectric constant ≈ 80). The acceptor water molecule is best represented implicitly using the polarizable continuum model (PCM) method; in this case, the p*K*_a_ value of the deprotonation site of the Mn_4_CaO_5_ cluster can be calculated, whereas the p*K*_a_ difference between the deprotonation site and the adjacent proton-acceptor water molecule cannot be calculated directly. On the other hand, in the presence of the PSII protein environment, proton release occurs along the H-bond between the ligand water molecule and the fixed acceptor group (i.e., fixed dipole with a low dielectric constant << 80) (Fig. 4b). The acceptor group is represented explicitly based on the crystal structure; in this case, the energy barrier, which is associated with the p*K*_a_ difference between the deprotonation site and the acceptor group^31^, can be calculated based on the potential-energy profile of the H-bond, whereas the p*K*_a_ value of the deprotonation site of the Mn_4_CaO_5_ cluster cannot be calculated directly. Note that only when the acceptor groups are always the same for all deprotonation sites (e.g., H_2_O), the p*K*_a_ values may be calculated from the p*K*_a_ difference [e.g., the difference from p*K*_a_(H_2_O/H_3_O^+^)]^31^. However, this is not the case for W1–W4 in the PSII protein environment, where the individual explicit acceptor groups already exist (e.g., D1-Asp61 for W1 and W446 for W2).

**Fig. 5:**
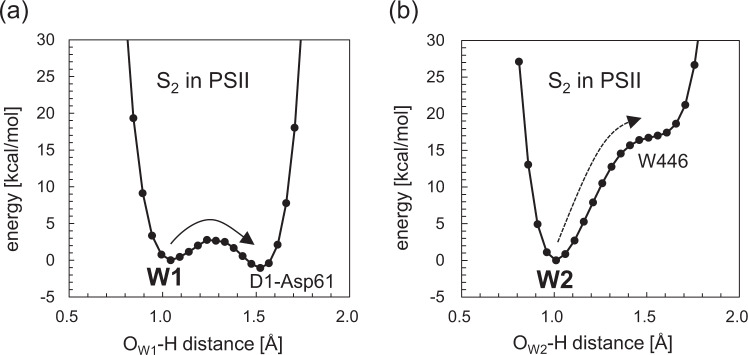
The potential energy profiles of the H-bonds in S_2_ [(Mn1, Mn2, Mn3, Mn4) = (III, IV, IV, IV)] in the PSII protein environment. **a** W1 and D1-Asp61. The isoenergetic proton transfer from W1 to D1-Asp61 indicates that p*K*_a_(W1) ≈ p*K*_a_(D1-Asp61). **b** W2 and the H-bond acceptor water molecule, W446. The energetically uphill proton transfer from W2 to W446 indicates p*K*_a_(W2) >> p*K*_a_(W1), i.e., deproton_a_tion of W2 is energetically less favorable than deprotonation of W1.

QM/MM calculations show that H_2_O at W1 forms a low-barrier H-bond with D1-Asp61 and the proton migrates towards the D1-Asp61 moiety in S_2_ (Fig. 5a), whereas H_2_O at W2 forms a standard H-bond with an adjacent water molecule (W446, Fig. 4b) and the proton is localized at the W2 moiety, i.e., proton transfer from W2 to the acceptor H_2_O is energetically uphill (Fig. 5b). This suggests that p*K*_a_(W1) is significantly lower than p*K*_a_(W2) in the PSII protein electrostatic environment and W2 cannot release the proton as more deprotonatable W1 exists at the Mn_4_CaO_5_ moiety in the PSII protein environment^32^. The results are consistent with W2 being H_2_O in S_1_ and S_2_ based on FTIR spectra and theoretical calculations by Nakamura and Noguchi^8^.

In summary, p*K*_a_(W1) ≈ p*K*_a_(W2) << p*K*_a_(W3) ≈ p*K*_a_(W4) in the Mn_4_CaO_5_ cluster in water (Table 2). p*K*_a_(W2) is only marginally (~1 p*K*_a_ unit) lower than p*K*_a_(W1) in the absence of the PSII protein environment (Table 2) if the Mn_4_CaO_5_ cluster is defined as shown in Fig. 1, which may be a basis of why electron paramagnetic resonance (EPR) signals were often interpreted based on simplified theoretical models with OH^−^ at W2 (e.g., refs. ^10,22,23^). p*K*_a_(W1) is significantly lower than p*K*_a_(W2) if the Mn_4_CaO_5_ cluster includes the second sphere ligand residues, D1-Asp61 and CP43-Arg357 (Table 3, Fig. 4a). Thus, as p*K*_a_(W1) and p*K*_a_(W2) depend strongly on the definition of the Mn_4_CaO_5_ region, p*K*_a_(W1) and p*K*_a_(W2) in water (in the absence of the protein environment) do not provide any clue to understanding the deprotonation sites in the physiological S-state transitions. The potential energy profiles of the H-bonds show that in the presence of the PSII protein environment in S_2_ Fig. 4b), H_2_O at W1 forms a low-barrier H-bond with D1-Asp61 and proton transfer is barrier-less (Fig. 5a)^26^, whereas H_2_O at W2 forms a standard H-bond with the adjacent H_2_O and proton transfer is energetically uphill (Fig. 5b)^32^. These suggest that p*K*_a_(W1) ≈ p*K*_a_(D1-Asp61) << p*K*_a_(W2) in PSII. As far as W1 exists, W2 can never release the proton (i.e., not OH^−^) in PSII.

## Methods

### p*K*_a_ calculation

In the deprotonation reaction of the protonated state (AH) to deprotonated state (A^–^) in water, p*K*_a_ is defined as

2 $${\mathrm{p}}K_{\mathrm{a}} = \frac{{\Delta G_{{\mathrm{water}}}}}{{2.303\,RT}},$$

where *ΔG*_aq_ is the free energy difference between (AH) and (A^–^ + H^+^) (i.e., Δ*G*_water_ = *G*_water_(A^–^) – *G*_water_(AH) + *G*_water_(H^+^)), *R* is the gas constant, and *T* is the temperature. Δ*G*_water_ can also be approximated as

3 $$\Delta G_{{\mathrm{water}}} = k\Delta E_{{\mathrm{water}}} + C,$$

where *k* is the scaling factor, Δ*E*_water_ is the energy difference between AH and A^–^, which can be calculated using a quantum chemical approach with the PCM method, and *C* is the constant (simple p*K*_a_ estimation with energy of the optimized geometry scheme^33^). If the p*K*_a_ values of molecules are obtained at the same temperature, Eq. (2) can be written into Eq. (4) using the Eq. (3) as

4 $${\mathrm{p}}K_{\mathrm{a}} = k^{\prime}\Delta E_{{\mathrm{water}}} + C^{\prime},$$

where *k*′ is the scaling factor and *C′* is constant. To determine *k*′ and *C′*, we calculated Δ*E*_water_ for 23 hexa-aqua metal complexes whose experimentally measured p*K*_a_ values are reported^21,34^.

### Hexa-aqua metal complex

The optimized geometry of the protonated hexa-aqua metal complex was obtained, using the restricted or unrestricted density functional theory with the B3LYP functional. The CSDZ* basis set was used for lanthanides except for La, the ERMLER2* basis set for actinides, and the LACVP* basis set for all other atoms. The spin states were consistent with previous studies by Galstyan et al.^34^. The optimized geometries of the deprotonated hexa-aqua metal complexes were obtained, fixing the torsion angles to prevent OH^−^ from forming an H-bond with other ligand H_2_O molecules. However, the H-bond formation between the ligand OH^−^ and H_2_O molecules could not be avoided for Ca^2+^, Zn^2+^, Cd^2+^, Dy^3+^, Th^4+^, Pa^4+^, U^4+^, Np^4+^, and Pu^4+^, which were excluded from the present study. It should be noted that by adding a few external H_2_O molecules to the ligand OH^−^ moiety, the H-bond formation between the ligand OH^−^ and H_2_O molecules could be avoided without fixing the torsion angles. It was reported that the p*K*_a_ values for hexa-aqua metal complexes in water did not differ significantly when calculated by fixing the torsion angles or adding a few external H_2_O molecules^34^, probably because the shape of the hexa-aqua metal complex is symmetrical. However, this does not hold true for W1–W4 in the Mn_4_CaO_5_ cluster whose shape is not symmetric. Adding a few external H_2_O molecules to the ligand OH^−^ moiety causes structural changes with respect to the original coordination geometry of the PSII crystal structure. In addition, explicit H_2_O water molecules (i.e., fixed dipole) form a specific H_2_O cluster (i.e., the dielectric constant << 80) at the deprotonatable ligand moiety, which does neither represent bulk water (i.e., the dielectric constant ≈ 80) nor provide the relevant p*K*_a_ values. Based on these, the torsion angles were fixed to obtain the optimized geometry for p*K*_a_ calculations in the present study. Using the optimized geometries, the energy difference (Δ*E*_water_) between the protonated and deprotonated states of hexa-aqua metal complexes were calculated with PCM method, using the Jaguar program^35^.

### Mn_4_CaO_5_ cluster

The optimized geometry of the Mn_4_CaO_5_ cluster in the PSII protein environment was obtained as follows: the atomic coordinates of PSII were taken from the X-ray structure of PSII monomer unit “A” of the PSII complexes from *Thermosynechococcus vulcanus* at a resolution of 1.9 Å (PDB code, 3ARC)^29^. Atomic partial charges of the amino acids were adopted from the all-atom CHARMM22^36^ parameter set, respectively. D1-His337 was considered to be protonated^8^. We employed the electrostatic embedding QM/MM scheme, in which electrostatic and steric effects created by a protein environment were explicitly considered, and we used the Qsite^37^ program code. We employed the unrestricted DFT method with the B3LYP functional and LACVP* basis sets. To analyze the Mn_4_CaO_5_ geometries and the H-bond potential-energy profiles, the QM region was defined as the Mn_4_CaO_5_ cluster (including the ligand side-chains of D1-Asp170, D1-Glu189, D1-His332, D1-Glu333, D1-Asp342, CP43-Glu354, the ligand carboxy-terminal group of D1-Ala344, and the ligand water molecules, W1–W4), the Cl-1 binding site (Cl-1, W442, W446, and the side-chains of D1-Asn181 and D2-Lys317), and the second-sphere ligands (side-chains of D1-Asp61 and CP43-Arg357). Specifically, the coordinates of the heavy atoms in the surrounding MM region were fixed at their original X-ray coordinates, while those of the H atoms in the MM region were optimized using the OPLS2005 force field. All of the atomic coordinates in the QM region were fully relaxed (i.e., not fixed) in the QM/MM calculation. All of the H-bond partners were included in the QM region. The cluster was considered to comprise ferromagnetically coupled Mn atoms, where the total spin *S* = 15/2 in S_0_, 14/2 in S_1_, and   13/2 in S_2_. The resulting Mn oxidation states (Mn1, Mn2, Mn3, Mn4) were (III, IV, III, III) in S_0_, (III, IV, IV, III) in S_1_, (III, IV, IV, IV) in open-cubane S_2_, and (IV, IV, IV, III) in closed-cubane S_2_. It should be noted that the difference in *S* (e.g., *S* = 1/2 in S_2_^38^, high, low, ferromagnetic, and antiferromagnetic) did not affect the values; e.g., (i) the resulting geometry^39,40^, (ii) the potential energy profile of proton transfer^26^, (iii) the redox potential of each Mn site^41^, and (iv) the p*K*_a_ values for the ligand water molecules W1–W4 in the absence of the protein environment (see Supplementary Table 2). To obtain the potential energy profiles of the O…H^+^…O bond, the QM/MM optimized geometry was used as the initial geometry. The H atom under investigation was moved between the two O moieties by 0.05 Å, after which the geometry was optimized by constraining the distance between O–H^+^ and H^+^–O distances, and the energy was calculated. This procedure was repeated until the H atom reached the O moieties.

In the absence of the PSII protein environment (i.e., in vacuum), the QM/MM-optimized geometry was re-optimized, using the unrestricted density functional theory with the B3LYP functional and LACVP* basis sets and fixing the torsion angles to maintain the overall shape of the less stable complex. The QM region was defined as either the Mn_4_CaO_5_ cluster (including the ligand side-chains of D1-Asp170, D1-Glu189, D1-His332, D1-Glu333, D1-Asp342, CP43-Glu354, the ligand carboxy-terminal group of D1-Ala344, and the ligand water molecules, W1–W4) or the Mn_4_CaO_5_ cluster and the second-sphere ligands (side-chains of D1-Asp61 and CP43-Arg357). Using the optimized geometries, the energy difference (Δ*E*_water_) between the protonated and deprotonated states of the Mn_4_CaO_5_ cluster were calculated with the PCM method, using the Jaguar program^35^.

## Supplementary information

Peer Review File

Supplementary Information

## Data Availability

All data generated or analyzed during this study are included in this article (and its Supplementary Information files).
