# Supplementary material for: pKa of the ligand water molecules in the oxygen-evolving Mn4CaO5 cluster in photosystem II
Source: Commun Chem. 2020 Jul 16;3:89. doi: 10.1038/s42004-020-00336-7 (PMC9814768; doi:10.1038/s42004-020-00336-7)
Supplement: Supplementary file 2 — Supplementary Information [file 42004_2020_336_MOESM2_ESM.docx]

Supplementary information

p*K*_a_ of the ligand water molecules in the oxygen-evolving Mn_4_CaO_5_ cluster in photosystem II

Keisuke Saito, Minesato Nakagawa, Hiroshi Ishikita*

**Supplementary Figure 1.** Experimentally measured p*K*_a_ values and calculated H_2_O/OH^−^ energy differences of hexa-aqua metal complexes in vacuum. Blue open circles for divalent (II) metals, green open squares for trivalent (III) metals, and red closed diamond for the tetravalent (IV) metal.

In the absence of water, i.e., in vacuum, the calculated energy difference (Δ*E*_vacuum_) between the protonated and deprotonated states of hexa-aqua metal complexes with the valences of II, III, and IV can be described as follows:

p*K*_a_ = 0.175 Δ*E*_vacuum_ [kcal/mol] – 60.1 + 19.1*n*, (Supplementary Equation 1)

where *n* is the total charge of the protonated (H_2_O) system (*n* = 1, 2, and 3).

**Supplementary Figure 2.** Rearrangement of the ligand coordination and formation of the H-bond network that proceeds from W2 via W3 toward W4 in the presence of OH^−^ at W4. This occurs when the geometry is fully relaxed (i.e., the torsion angles are not fixed).

**Supplementary Table 1.** Calculated (Calc.) and experimentally measured (Expl.) p*K*_a_ values of metal-centred aqua complexes. The p*K*_a_ values were calculated by (eq. S1) from Δ*E*_vacuum_ of metal-centred aqua complexes.

| metal ion | Calc. p*K*_a_ | Expl. p*K*_a_ ^a^ |
| --- | --- | --- |
| Zr^4+^ | 0.5 | −0.32 |
| Mn^3+^ (high spin) | 0.4 | 0.08 |
| Fe^3+^ (high spin) | 2.4 | 2.19 |
| Ti^3+^ | 3.0 | 2.20 |
| V^3+^ | 3.0 | 2.60 |
| Ru^3+^ (low spin) | 3.6 | 2.90 |
| Co^3+^ (low spin) | 2.0 | 2.92 |
| Rh^3+^ (low spin) | 3.9 | 3.40 |
| Cr^3+^ | 3.3 | 4.29 |
| Sc^3+^ | 4.3 | 4.30 |
| Lu^3+^ | 8.4 | 7.94 |
| Cu^2+^ | 6.3 | 8.0 |
| Y^3+^ | 7.1 | 8.34 |
| Pr^3+^ | 7.6 | 8.91 |
| La^3+^ | 8.9 | 9.03 |
| Fe^2+^ (high spin) | 9.9 | 9.50 |
| Co^2+^ (high spin) | 9.0 | 9.65 |
| Gd^3+^ | 8.5 | 9.78 |
| Ni^2+^ | 9.1 | 9.86 |
| Mn^2+^ (high spin) | 10.2 | 10.59 |
| Mg^2+^ | 9.7 | 11.41 |
| Sr^2+^ | 15.3 | 13.18 |
| Ba^2+^ | 17.1 | 13.36 |
| RMSD^b^ | 1.2 |  |

^a^ See Table 1. ^b^ Root mean square deviation of the calculated p*K*_a_ value from the measured p*K*_a_ value.

**Supplementary Table 2.** Calculated p*K*_a_ values of the ligand water molecules of the Mn_4_CaO_5_ cluster with different spin configurations in water (in the absence of the protein environment).

| S state | valence  (Mn1,Mn2,Mn3,Mn4) | spin configuration  (Mn1,Mn2,Mn3,Mn4) | W1 | W2 | W3 | W4 |
| --- | --- | --- | --- | --- | --- | --- |
| S_0_ | (III, IV, III,III) | (↑, ↑, ↑, ↑) | 11.3 | 10.1 | 17.7 | 16.4 |
|  |  | (↓, ↑, ↓, ↑) | 11.4 | 10.1 | 17.7 | 16.0 |
|  |  | (↑, ↓, ↓, ↑) | 11.3 | 10.3 | 17.5 | 16.9 |
|  |  | (↑, ↑, ↓, ↓) | 11.3 | 10.1 | 17.3 | 16.1 |
| S_0_  [O4-H]^a^ | (III, IV, III,III) | (↑, ↑, ↑, ↑) | 9.5 | 9.0 | 16.6 | 16.0 |
|  |  | (↓, ↓, ↑, ↑) | 9.5 | 8.8 | 16.6 | 15.6 |
|  |  | (↑, ↓, ↓, ↑) | 9.5 | 8.9 | 16.6 | 16.1 |
|  |  | (↑, ↓, ↑, ↓) | 9.5 | 9.0 | 16.6 | 16.0 |
| S_0_  [O5-H]^b^ | (III, IV, III,III) | (↑, ↑, ↑, ↑) | 10.0 | 8.5 | 16.1 | 15.4 |
|  |  | (↓, ↓, ↑, ↑) | 10.1 | 8.7 | 16.1 | 15.5 |
|  |  | (↑, ↓, ↓, ↑) | 9.9 | 8.6 | 16.1 | 15.4 |
|  |  | (↑, ↓, ↑, ↓) | 10.0 | 8.7 | 16.1 | 15.7 |
| S_1_ | (III, IV, IV,III) | (↑, ↑, ↑, ↑) | 10.2 | 9.0 | 15.6 | 15.1 |
|  |  | (↓, ↓, ↑, ↑) | 10.0 | 8.5 | 16.1 | 15.4 |
|  |  | (↑, ↓, ↑, ↓) | 10.1 | 8.7 | 16.1 | 15.5 |
|  |  | (↓, ↑, ↓, ↑) | 9.9 | 8.6 | 16.1 | 15.4 |
|  |  | (↑, ↑, ↓, ↓) | 10.0 | 8.7 | 16.1 | 15.7 |
| S_2_ [open]^c^ | (III, IV, IV,IV) | (↑, ↑, ↑, ↑) | 8.3 | 8.2 | 15.6 | 15.9 |
|  |  | (↑, ↑, ↓, ↓) | 8.1 | 8.2 | 15.7 | 16.1 |
|  |  | (↑, ↓, ↑, ↓) | 8.2 | 8.1 | 14.5 | 15.5 |
|  |  | (↑, ↓, ↓, ↑) | 8.2 | 8.1 | 15.3 | 15.7 |
| S_2_ [closed]^d^ | (IV, IV,IV,III) | (↑, ↑, ↑, ↑) | 9.7 | 7.1 | 14.2 | 15.6 |
|  |  | (↑, ↑, ↑, ↓) | 9.7 | 7.0 | 14.2 | 15.6 |

^a^ O4 is protonated. ^b^ O5 is protonated. ^c^ Open-cubane structure. ^c^ Closed-cubane structure.
